# Transcriptome assembly for a colour-polymorphic grasshopper (*Gomphocerus sibiricus*) with a very large genome size

**DOI:** 10.1186/s12864-019-5756-4

**Published:** 2019-05-14

**Authors:** Abhijeet Shah, Joseph I. Hoffman, Holger Schielzeth

**Affiliations:** 1 Institute of Ecology and Evolution, Friedrich Schiller University Jena, Dornburger Str. 159, 07743 Jena, Germany; 2 Department of Animal Behaviour, Bielefeld University, Morgenbreede 45, 33615 Bielefeld, Germany

**Keywords:** Insects, Orthoptera, Acrididae, Gomphocerinae, Transcriptome, Mitochondria, *Wolbachia*

## Abstract

**Background:**

The club-legged grasshopper *Gomphocerus sibiricus* is a Gomphocerinae grasshopper with a promising future as model species for studying the maintenance of colour-polymorphism, the genetics of sexual ornamentation and genome size evolution. However, limited molecular resources are available for this species. Here, we present a de novo transcriptome assembly as reference resource for gene expression studies. We used high-throughput Illumina sequencing to generate 5,070,036 paired-end reads after quality filtering. We then combined the best-assembled contigs from three different de novo transcriptome assemblers (Trinity, SOAPdenovo-trans and Oases/Velvet) into a single assembly.

**Results:**

This resulted in 82,251 contigs with a N50 of 1357 and a TransRate assembly score of 0.325, which compares favourably with other orthopteran transcriptome assemblies. Around 87% of the transcripts could be annotated using InterProScan 5, BLASTx and the *dammit!* annotation pipeline. We identified a number of genes involved in pigmentation and green pigment metabolism pathways. Furthermore, we identified 76,221 putative single nucleotide polymorphisms residing in 8400 contigs. We also assembled the mitochondrial genome and investigated levels of sequence divergence with other species from the genus *Gomphocerus*. Finally, we detected and assembled *Wolbachia* sequences, which revealed close sequence similarity to the strain pel wPip.

**Conclusions:**

Our study has generated a significant resource for uncovering genotype-phenotype associations in a species with an extraordinarily large genome, while also providing mitochondrial and *Wolbachia* sequences that will be useful for comparative studies.

**Electronic supplementary material:**

The online version of this article (10.1186/s12864-019-5756-4) contains supplementary material, which is available to authorized users.

## Background

One important goal of functional genomics is to establish links between genetic polymorphisms and phenotypic variation [1]. Recent developments in high-throughput sequencing technologies have greatly facilitate this endeavour. However, there are still major challenges inherent to genomic approaches for genotype-phenotype associations in non-model organisms [2]. One of these challenges is imposed by species with large genomes, as genome assemblies are difficult to construct for highly repetitive regions [3]. To some degree, this issue will be mitigated by the development of long-range sequencing technologies [4]. Nevertheless, we expect that genomic approaches for taxa with very large genomes will remain challenging for some time.

Transcriptomics offers an alternative to genomic approaches, as the size of the transcriptome does not scale linearly with genome size and most functional differences with phenotypic effects should be reflected in the transcriptome [5]. Not only do transcripts differ in their sequences, mirroring underlying coding DNA sequence differences, but quantitative analysis can also allow the assessment of regulatory variation influencing transcript abundance [6, 7]. One prerequisite for an analysis of transcript abundance is a high quality transcriptome assembly, as this acts as a reference against which short read RNA sequencing data can be mapped [8].

Grasshoppers from the family Acrididae (Orthoptera, Caelifera) encompass a large number of species, including several economically relevant species, but have been poorly studied molecularly, partly because of their often very large genomes [9]. The 1Kite project has set out to sequence 1000 insect transcriptomes, including 43 species of Orthoptera, but only two Acridids (http://1kite.org/downloads/1KITE_species.txt). This is surprising because the Acridids are the largest family of Orthopterans, comprising around half of all Orthopteran species [10]. The only Acridid transcriptomes published so far are for the desert locust *Schistocerca gregaria* [11], the migratory locus *Locusta migratoria* [12], the stripe-winged grasshopper *Stenobothrus lineatus* [13] and the bow-winged grasshopper *Chorthippus biguttulus* [14].

The two locusts, *Schistocera* and *Locusta*, belong to the subfamilies Cyrtacanthacridinae and Oedipodinae respectively and have moderately large genomes [9]. Both species are of great interest due to their involvement in pest outbreaks and remarkable phenotypic plasticity. However, true phase-polymorphism and swarming behaviour is rather unusual, even among Acridids [15]. From an evolutionary perspective, the Acridids are remarkable for another phenomenon, the taxonomically widespread occurrence of an apparently balanced green-brown polymorphism in body coloration [16, 17].

The genera *Stenobothrus* and *Chorthippus* are representatives of the Gomphocerinae, a large subfamily of Acididae with particularly large genomes [9] and widespread green-brown polymorphisms, but no locust-like swarming behaviour. Of those two, only *Chorthippus biguttulus* is green-brown polymorphic.

**Fig. 1:**
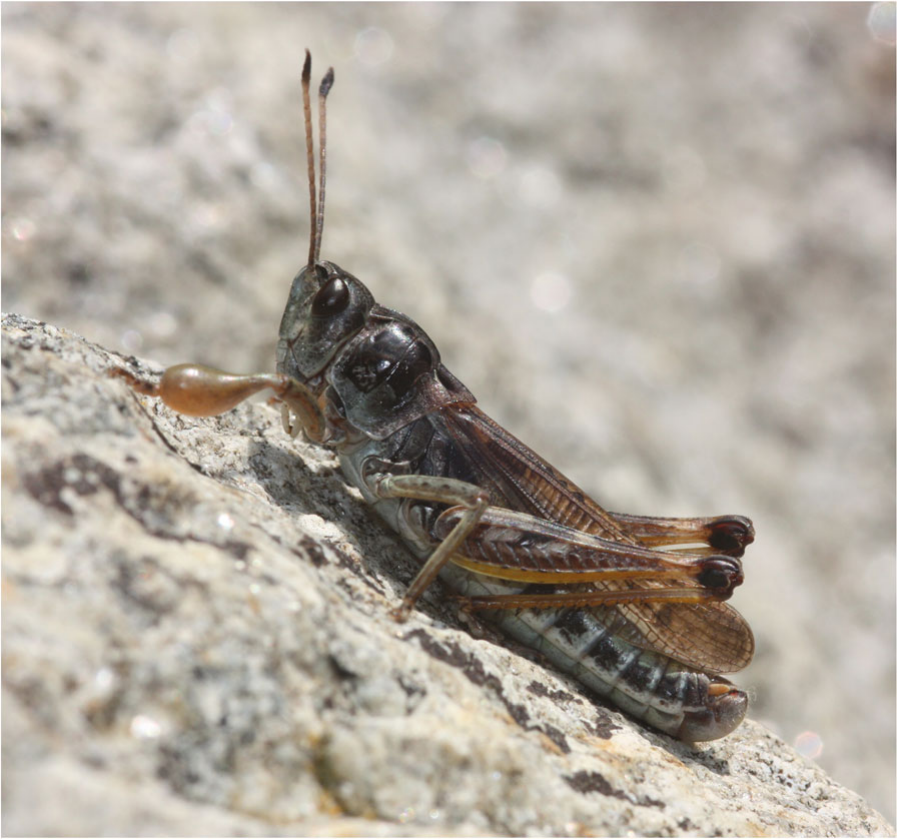
The club-legged grasshopper *Gomphocerus sibiricus*, an alpine-dwelling species exhibiting prominent sexual dimorphism of the front leg. Photo credit: Holger Schielzeth

Here, we present a de novo transcriptome assembly for the club-legged grasshopper *Gomphocerus sibiricus*, an alpine-dwelling species that is unusual for its striking sexual dimorphism in front leg morphology [18] (see Fig. 1) and is also characterized by the widespread occurrence of a balanced green-brown polymorphism [17]. The green-brown polymorphism has been found to be unrelated to rearing conditions [19] and recent evidence from the laboratory suggests that it follows a simple Mendelian mode of inheritance (unpublished data). Biliverdin is the dominant green pigment in Orthopterans [20] and it is likely that the biliverdin synthesis pathway plays an important role in the determination of body colour. However, the specific genes involved in producing the green-brown polymorphism are unknown in this species as well as in grasshoppers in general. Transcriptomic analysis thus offers a promising route for uncovering the genetic basis of this eco-evolutionarily relevant trait in club-legged grasshoppers as well as potentially in other species.

We assembled cDNA sequencing data from five *G. sibiricus* individuals including both males and females as well as specimens of the two colour morphs. In order to generate the most exhaustive assembly possible, we employed three different commonly used assemblers and combined the resulting assemblies into a single high-quality transcriptome assembly [21, 22]. We then compared our assembly to other published grasshopper assemblies and mined the transcripts for the presence of candidate genes involved in biliverdin pigmentation pathways. Additionally, we analysed the expression profile of the mitochondria and compared this to data from closely related species. Finally, having also found evidence for the presence of the parasitic microbe *Wolbachia* in our sample, we decided to assemble and analyse the *Wolbachia* strain present in this grasshopper species.

## Results

### Transcriptome assembly assessment and completeness metrics

We assembled a draft *G. sibiricus* transcriptome using three assemblers, followed by pooling of assemblies and removal of duplicates. The final draft assembly comprised 82,251 contigs, 21,347 of which contained open reading frames (ORFs). The TransRate transcriptome assembly metric was 0.325, which is similar to or better than most previously published orthopteran transcriptomes (Table 1). In order to further assess the quality of the transcriptome assembly, we constructed a kernel density plot of contig lengths versus the average depth of sequences mapping to those contigs (Additional file 1: Figure S1). Overall, the mapping rate using both read datasets was 96.72% (97.02% when using normalised reads, read mapping statistics for non-rRNA reads only are available in Additional file 7: Table S3) with BWA under default settings. The mean (median) contig length was 1057 bp (718 bp) and the mean (median) coverage was 52.6x (11x), indicating that we were able to assemble relatively complete contigs.

**Table 1:** TransRate assembly metrics and BUSCO completeness assessment for *Gomphocerus sibiricus* (this study) in comparison to the other two Acridid transcriptomes published (*Stenobothrus lineatus*, [13], *Chorthippus biguttulus*, [34]). Higher TransRate assembly scores indicate better quality assemblies. BUSCO completeness assessment was conducted using the insect ortholog database (orthoDB9)

TransRate metrics	*Gomphocerus sibirucus*	*Stenobothrus lineatus*	*Chorthippus bigullutus*
Number of contigs	82,251	57,778	67,733
Number of contigs with ORF	21,347	12,717	30,018
N50 of contig length	1357	1207	1246
Length of longest contig	43,026	22,561	34,437
Length of shortest contig	301	200	600
Proportion of read fragments mapped	0.88	0.70	0.51
Proportion of good read pairs mapping	0.82	0.63	0.43
TransRate assembly score	0.325	0.162	0.106
BUSCOs (Number of BUSCO units found)			
Complete BUSCOs	1405	1337	1489
Complete and Single-Copy BUSCOs	1093	1244	1323
Complete and duplicated BUSCOs	312	93	166
Fragmented BUSCOs	137	142	99
Missing BUSCOs	116	179	70
Total BUSCOs searched	1658	1658	1658

### Transcriptome annotation and GO classification

Over 87% of the contigs were annotated using InterProScan 5, BLASTx (non-redundant protein database) and the *dammit!* annotation pipeline. The InterProScan analysis resulted in 1,588,733 matches from 72,132 annotated sequences, with 337,258 assigned membrane-bound protein signatures. Furthermore, 109,437 GO terms were assigned to 39,794 transcripts. A BLASTX search of transcripts was run against the non-redundant (nr) protein database which yielded 12,436,946 hits from 31,527 transcripts. Around 78% (24,568) of these transcripts were assigned to insects, and the next largest fraction (3.8%) was assigned to the Arachnida (Additional file 2: Figure S2). The *dammit!* annotation pipeline provided us with 363,218 annotations from 44,488 transcripts. Of these, 41,535 transcripts were annotated by both the InterProScan 5 and *dammit!* Pipelines (Additional file 5: Table S1).

The annotated draft transcriptome was classified into three main categories of GO components: cellular components, molecular function and biological processes (Additional file 3: Figure S3). Of these, 3617 (9.1%) were classified as cellular component, 26,974 (67.8%) as molecular function and 9203 (23.2%) as biological process. Furthermore, GO terms associated with accumulation of pigment and haeme biosynthesis (GO:0043473 pigmentation; GO:0006783, GO:0006784 haeme; GO:0048034 haeme-O and haeme oxygenase decyclizing activity) were found amongst these annotations. Additionally, we found several relevant InterPro terms: IPR015118 (5-aminolevulinate synthase presequence), IPR010961 (tetrapyrrole biosynthesis of 5-aminovulenic acid), IPR002051 (haeme oxygenase) and KEGG reference: 00860 + 1.14.14.18 (biliverdin producing haeme oxygenase) in our annotations. This provides a starting point for investigating differential gene expression in relation to the green-brown colour polymorphism in future studies.

### SNP calling and estimation of minor all frequencies (MAF)

**Fig. 2:**
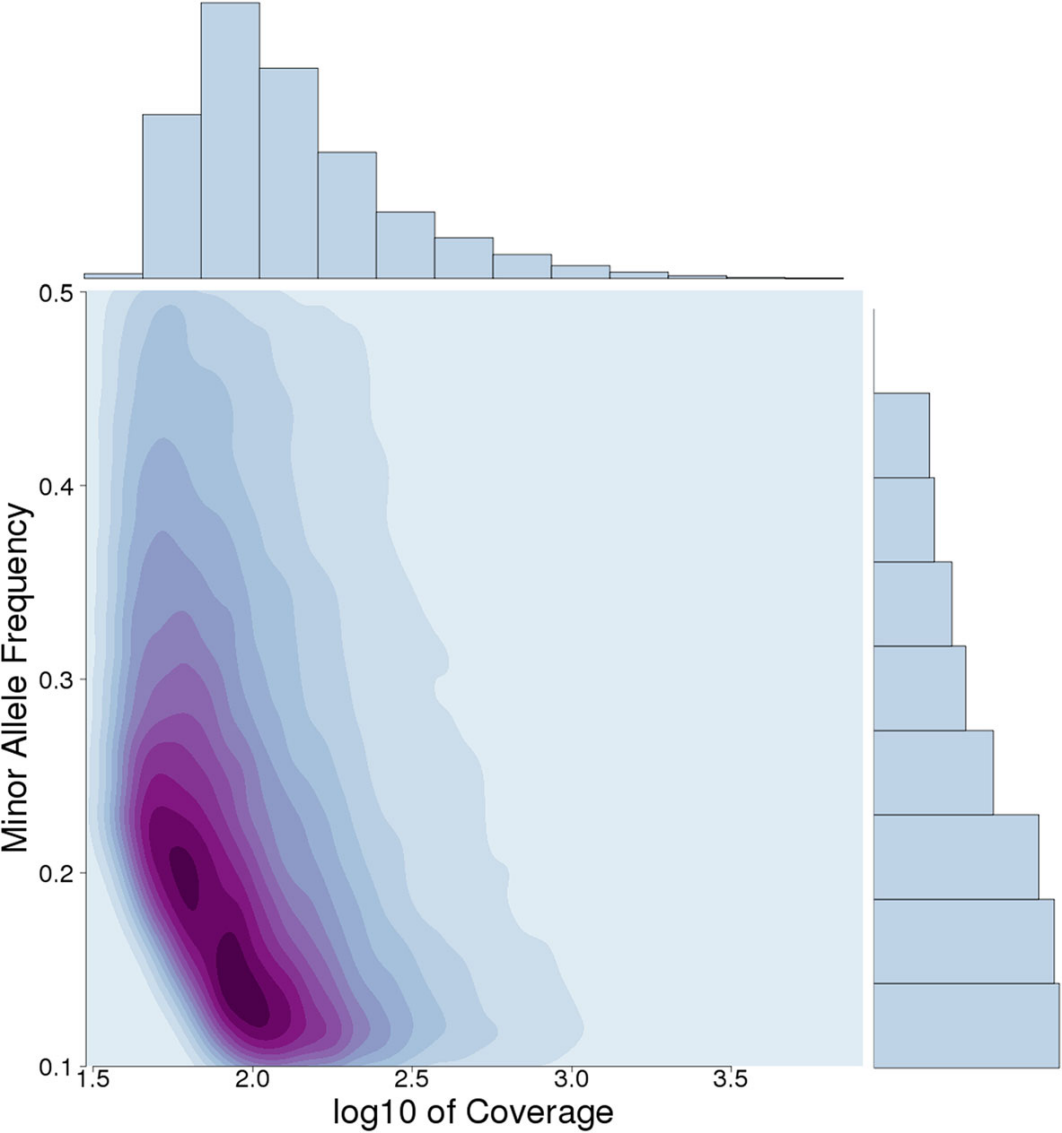
A two-dimensional kernel density plot showing minor allele frequency (MAF) plotted against log10 sequence coverage. The dark purple regions indicate higher densities, whereas the light blue regions indicate lower densities. Marginal histograms show frequency distributions of minor allele frequency (top axis) and coverage (right axis)

We found 76,221 SNPs in 84,00 contigs with mean of 9.07 variants per contig. A contour plot shows that minor allele frequencies (MAFs) peak between 0.12 and 0.2, while rarer variants could only be detected with increasing depth of sequencing coverage (Fig. 2).

### Scanning for expressed mitochondrial genes in assembled transcripts

**Fig. 3:**
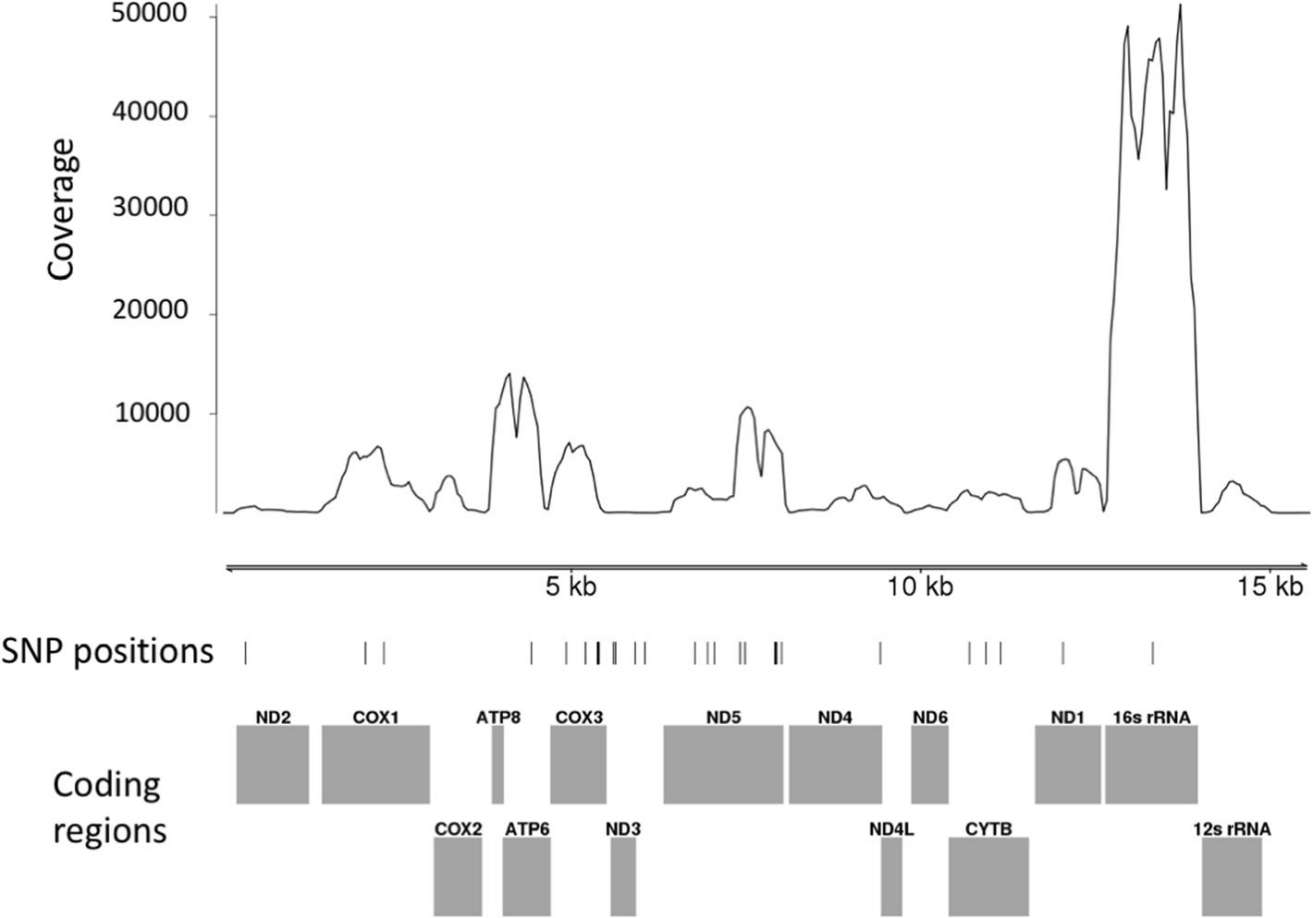
Transcripts from *Gomphocerus sibiricus* mapping to the reference mitochondrial genome assembly. The short vertical lines indicate positions of putative SNPs. The grey shaded blocks indicate protein and rRNA coding regions

*G. sibiricus* mitochondrial sequences were also found in the assembled transcripts, and their coverage depth was estimated. An alignment of the assembled draft transcripts against the published mitochondrial genome [23] shows that we could recover large multi-gene segments. The published mitochondrial genome of *G. sibiricus* (refseq: NC_021103.1) consists of a standard set of 13 protein coding genes and two ribosomal RNA genes (16 s and 12 s ribosomal sub-units). As expected, our contigs mapped to the reference across the full mitochondrial genome with particularly high coverage over the 16 s and 12 s ribosomal RNA genes (Fig. 3).

The distance matrix of the mitochondrial genome assembled from this study and four closely related taxa provides an overview of the amount of sequence divergence within the genus *Gomphocerus*. As expected, the lowest divergence was found between our European *G. sibiricus* population and the Asian population of the same species (divergence 1.6%, Additional file 6: Table S2), while there was slightly more divergence with the Asian congeneric species *G. licenti* and *G. tibetanus*, and the largest divergence was found in the comparison with *G. rufus* (which is usually placed within the genus *Gomphocerippus*). This analysis also uncovered two unexpected patterns. First, *G. sibiricus* was found to be less divergent from *licenti* than from *tibetanus*, even though the latter is sometimes considered a subspecies of *G. sibiricus* [10]. Second divergence estimates with *licenti* / *tibetanus* were lower than with the published *G. sibiricus* sequence from Central Asia.

### Detection of Wolbachia Pel wPip strain sequences

**Fig. 4:**
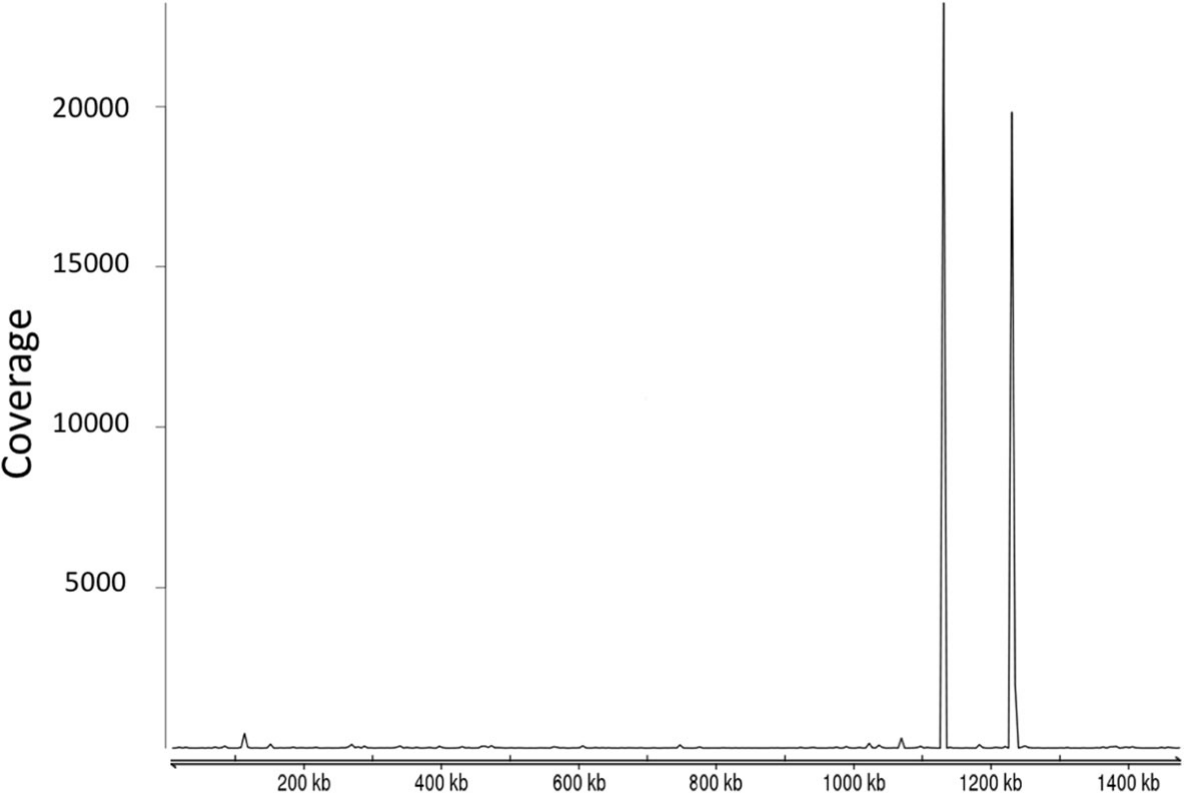
Coverage of the *Wolbachia* genome estimated by mapping *G. sibiricus* transcripts against the pel wPi strain of *Wolbachia*. The major peaks around positons 1,136,000 bp and 1,236,0000 bp correspond to the 16 s and 23 s rRNA coding regions

We detected the endosymbiont *Wolbachia* in our RNA sequencing data. For strain determination, we mapped our *Wolbachia* assembly to all the *Wolbachia* genomes available on GenBank (8280 assemblies, retrieved 30/11/2017) using *BWA* aligner. Our *Wolbachia* strain showed the best mapping to strain wPip, which was originally described from the *Culex quinquefasciatus* Pel genome [24]. Mean read coverage was quantified using a sliding window of 250 bp (Fig. 4). The coverage of *G. sibiricus* transcripts suggests ample uniform coverage, with two major peaks close to genome positions 11,360,000 and 12,360,000, which correspond to 16 s and 23 s ribosomal RNA respectively. Additionally, a table containing the transcripts which have the top BLASTX hits to *Wolbachia* are provided in table in an Additional file 8.

## Discussion

We here present a high quality transcriptome assembly for *G. sibiricus*, a species of grasshopper with a large genome that is of particular evolutionary interest because it features a green-brown polymorphism that is shared with many other Orthopterans and seems to be maintained in natural populations by balancing selection [17]. Assembly quality statistics from BUSCO and TransRate demonstrate that the assembly is of similar quality to the only two available assemblies for the subfamily Gompocerinenae, which are also characterized by large genome sizes (the estimated genome size of *G. sibiricus* is approximately 8.75 Gb) [25]. Our draft transcriptome provides a resource for future studies of differential gene expression, which should ultimately help to improve our understanding of the genetic basis of colour morph determination. Furthermore, our mitochondrial and *Wolbachia* sequence assemblies provide material for phylogenetic and comparative analyses.

Previously, a comparison of Gomphocerinae mitochondrial genomes [23] suggested that *G. sibiricus* is more closely related to *G. licenti* than to *G. tibetanus*, although *tibietanus* is sometimes listed as a subspecies of *sibiricus*, while *licenti* is considered a separate species [10]. Our analysis with an independent sample of *sibiricus* confirms the results of Zhang et al. [23], as we also found less divergence from *licenti* than from *tibetanus*. Despite the greater geographical distance, however, we find our sample to be closer to *licenti* / *tibetanus* than the previously published *G. sibiricus* mitochondrial sequence from China. This may be related to the quality of the assembly (erroneous assemblies will tend to reduce similarity) or it could potentially reflect a yet unexplored and surprising colonization history from Central Asia to the mountains in Europe.

We also found the first evidence that *G. sibiricus* is infected by *Wolbachia*, a microbial parasite that is known to manipulate host reproductive biology and can cause horizontal gene transfer [26, 27]. Our finding is not entirely unexpected given that many insect species are known to be infected by *Wolbachia* [28]. Moreover, *Wolbachia* infections have recently been reported in other grasshopper species including *Chorthippus parallelus* [29, 30] and *Podisma saporensis* [31]. *Wolbachia* causes cytoplasmic incompatibility in *C. parallelus* [30] and is suspected to play a leading role in causing hybrid dysfunction in *P. saporensis* [31]. Our draft transcriptome assembly therefore provides a basis for future studies investigating the evolutionary and ecological significance of *Wolbachia* infections in *G. sibiricus*.

As we were able to annotate the majority of our assembled transcripts, our assembly provides a useful tool for investigating the genetic basis of complex traits such as pigmentation. Our GO annotations allowed us to identify putative candidate genes for investigating the mechanism of colour morph determination. We demonstrated the presence of multiple transcripts that are putatively involved in pigmentation, more specifically, in the porphyrin and chlorophyll metabolism pathway (KEGG ko00860) and in haeme oxygenase 2 (biliverdin-producing, HMOX2) [32]. Furthermore, we found indirect evidence for precursors and other metabolites involved in pigmentation pathways, possibly reflecting the lack of available metabolic and biochemical resources and the significant phylogenetic distance of *G. sibiricus* from well-annotated model organisms. Importantly, the GO and InterPro evidence for transcripts involved in haeme and haeme-O complex metabolism (GO:0006783, GO:0006784, GO:0048034, IPR015188, IPR010961) suggests that components for a plausible mechanism to metabolize green pigments from plant sources exist in this species.

Our transcriptome assembly of *G. sibiricus* combines the best assembled transcripts from three different de novo transcriptome assemblers (with multiple k-mer assemblies) to detect, capture and assemble transcripts. Recently, Smith-Unna et al. [33] investigated de novo transcriptome assembly quality from 155 previously published transcriptome assemblies. They found that assemblies generated by individual assemblers yielded relatively low TransRate scores, but when multiple assemblies were combined, reduced and filtered, they yielded reasonably representative collections of sequenced read fragments. According to this particular quality metric, our draft transcriptome assembly for *G. sibiricus* lies in the approximate upper 70th percentile of the surveyed published transcriptomes, suggesting that the quality of our assembly is well above average. In our case, the combination of different assemblers was key to effective transcriptome assembly.

## Conclusions

In conclusion, we generated a high quality transcriptome assembly for *G. sibiricus*, a species with a large genome and limited available molecular resources. Not only will our study provide a solid foundation for studying the genetic basis of green-brown colour dimorphism in *G. sibiricus*, but we also generated resources that should facilitate future studies of mitochondrial genome evolution and *Wolbachia* infections of Orthopterans.

## Methods

### Sample collection

Individuals of *Gomphocerus sibiricus* were collected from a field site at 1800–2000 m near Sierre (Valais, Switzerland) in 2013. Five individuals of their laboratory-reared offspring were selected for RNA sequencing. This included one imago brown female, one imago green female, one imago brown male, one imago green male and one last-instar green female. Sexes, colour morphs and developmental stages were mixed in order to achieve a sufficient representation of the species’ transcriptome.

### RNA extraction, cDNA library preparation and spike-in normalisation (step 1)

Total RNA was extracted using an innoPrep RNA kit (Analytik Jena, Jena, Germany), followed by quality control and quantification using the RNA 6000 Nano LabChip kit with the Bioanalyzer 2100 (Agilent, Santa Clara, CA, USA). The samples were pooled before cDNA library for transcriptome sequencing was constructed. In order to maximize sequencing efforts, the poly-(A)-containing mRNA was purified from total RNA using poly-(T) oligo-attached magnetic beads. This was followed by cDNA library construction, which was prepared and sequenced by the Center for Biotechnology at Bielefeld University on the Illumina MiSeq platform (San Diego, California, USA) with a maximum read length of 300. Next, an aliquot of the cDNA library was sent to a commercial facility for standard ERCC spike-in normalization, followed by an additional round of sequencing using the same protocol, to ensure that sequencing efforts were not only concentrated on highly abundant sequences [34]. The normalised library was used for transcriptome assembly, while the non-normalised library was used for read mapping and transcript confirmation.

### Pre-processing and sequence quality control and k-mer filtering (step 2)

All pair-end reads were processed and trimmed to a maximum length of 300 bp to remove low-quality bases. The resulting reads were assessed for quality using FastQC (version 0.11.15). Sequence quality and adapter trimming was performed using the trimmomatic tool (version 0.36) [35], with a four base sliding window. Bases below a phred quality score of 15 were removed as were reads with a sequence length below 150 using the setting ‘2:30:10 LEADING:3 TRAILING:3 SLIDINGWINDOW:4:15 MINLEN:150’. Furthermore, the reads were in silico normalized using Khmer (version 2.0) following the authors’ recommended protocol for trimming reads with variable coverage (recipe 7) [36]. Additional artefact filtering was carried out using the FASTX toolkit (version 0.0.14). This resulted in a final read dataset of 5,070,036 paired-end reads with a median read length of 249 bp for the normalised read dataset.

### Transcriptome assemblies (step 3)

**Fig. 5:**
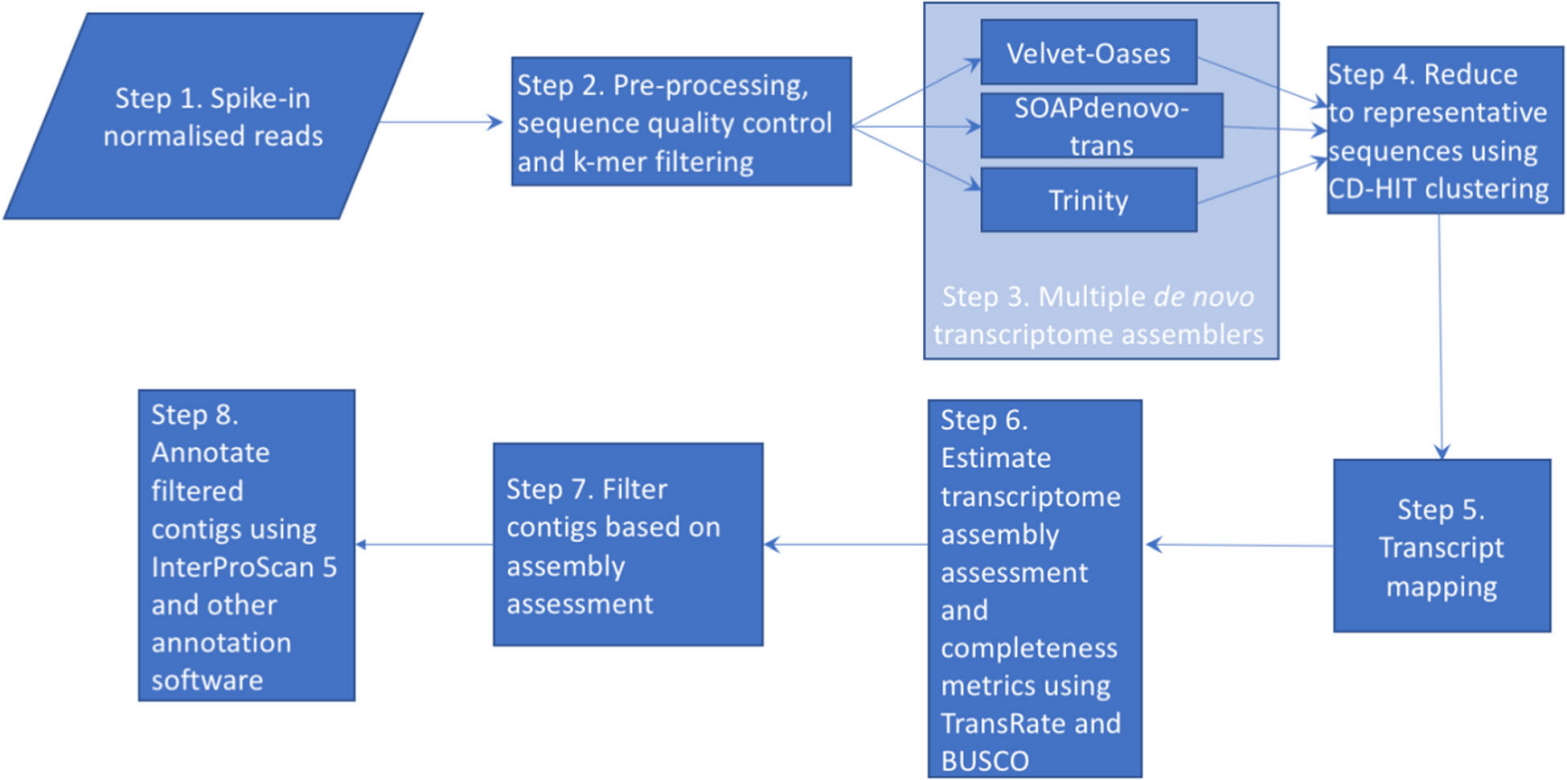
Flow chart showing a summary of the de novo assembly procedure and downstream analyses

We assembled the *G. sibiricus* transcriptome using the de novo transcriptome assembly packages SOAPdenovo-trans [37], Trinity [38] and Oases-Velvet [39]. An overview of this process is given in Fig. 5. First, we used SOAPdenovo-trans (version 1.03) to build 54 de novo assemblies with k-mer values of 21 to 127 (in steps of 2) with an average insert size of 475 bp, using -F and -L 300 command parameters to set filling the gaps in scaffolds and shortest contig length for scaffolding to 300 bp. Second, we used Trinity (release v2.2.0) with the default settings, the minimum transcript length set to 300 bp, and with *in-silico* read normalization with default settings. Third, Oases-Velvet (version 0.2.09) was used to build eighteen de novo assemblies with k-mer values of 21 to 55 (in steps of 2) with an insert length of 475 bp and the minimum transcript length set to 300 bp. Assemblies with higher k-mer values were not possible due to memory constraints.

### Multi-assembly merging and duplicate contig reduction (step 4)

All of the assemblies were collated into a single file and followed by removal of duplicate and redundant contigs. CD-HIT (cd-hit-est, version 4.6) [40], a greedy incremental clustering algorithm, was used to remove duplicate and highly similar sequences (sequence similarity greater than 90% identity) to generate representative sequences of all of the assembled transcripts from all of the assemblies. This approach allowed us to capture as many unique transcripts as possible from three different de novo transcriptome assemblers.

### Transcript mapping and quantification (step 5)

The quality filtered reads were mapped to our de novo transcriptome assembly and the number of mapping reads per transcript was quantified using BWA (version 0.712-r1039) [41]. Per contig and per base coverage was calculated using BBMap [42] and SAMtools (version 1.4) [43].

### Assessment of assembled transcriptome and contig filtering (step 6 and 7)

We assessed the quality of the de novo assembly using the TransRate package (version 1.0.3) [33]. This assesses transcriptome assembly accuracy using read and contig data and estimates individual contig and overall assembly quality. Contigs that scored low on the contig assessment criteria (contig score components: S(C_seg_) and S(C_cov_), with default thresholds as suggested by TransRate), were filtered out. We then compared our empirical assembly metrics to the published transcriptomes of two other Gomphocerine species for which transcriptome assemblies have been published (*Stenobothrus lineatus* [13] and *Chorthippus biguttulus* [44]). Assembly completeness was assessed with Benchmarking Universal Single-Copy Orthologs (BUSCO, version 2.0) [45]. BUSCO defines a set of core genes for a given group or lineage and uses these genes as proxy for minimum completeness assuming that these genes should encode a large set of core genes. For this analysis, we used the genome and transcriptome completeness assessment tool in transcriptome assessment mode with the insect lineage database (insecta_orthoDB9, created 13/02/2016).

### Annotation and sequence analysis (step 8)

After assembly and assembly assessment, we annotated and analysed the assembly. InterProScan 5 is a widely used sequence analysis framework to search for various analytical signatures from different databases including the InterPro protein database. The draft transcript sequence signatures were scanned against InterPro’s signatures (version 5.22) [46]. TMHMM, SignalP, TIGRFAMs, Prosite, Panther, PFam, PIRSF, CDD, COILS, and Gene3D applications were selected to run with the transcripts using the InterScanPro 5 framework. Additional annotation was generated using the *dammit!* annotation pipeline (https://github.com/dib-lab/dammit/). This pipeline was set to use the Pfam, Rfam, OrthoDB, uniref90 and BUSCO arthropoda databases in the ‘--full’ mode. All assembled contigs were also searched against the BLASTx non-redundant protein database (Nr) and results with expect values (e-values) below 10^− 6^ were reported. To visualize the BLAST profile of the assembly, we selected the best matches (based on best bit-scores) from every transcript and plotted the counts of taxonomic classes found.

### SNP calling and estimation of minor allele frequencies

We detected SNPs from the transcriptome by mapping the quality-filtered reads to the final set of assembled transcripts using the *BWA* aligner (version 0.712-r1039) [47] (using default settings) followed by variant detection with VarScan (version 2.4.2) [48] with minimum coverage set to 30, a minimum expected variant frequency set to 0.1, and *p*-value threshold of 0.01. In order to visualize our variants with respect to sequencing depth, we estimated the two-dimensional kernel density of the minor allele frequency and plotted this as a contour plot.

### Searches for candidate green-brown pigmentation genes

Gene ontology terms (GO terms) associated with pigmentation (GO:0043473) and haeme-metabolism (GO:0004392, GO:0006784 and GO:0048034) were selected as possible candidates for genes associated with the observed green-brown dimorphism. Furthermore, InterPro and KEGG terms (IPR010961, IPR015118, IPR002051, KEGG00860 + 1.14.14.18) associated with haeme oxygenase (the pathway leading to biliverdin production) were also added to the annotation search. The presence of these GO terms illustrates that putative candidates for pigmentation are represented in our assembly.

### Analysis of the mitochondrial genome

We also assembled the mitochondrial transcriptome of *G. sibiricus*. Reads were mapped to a the reference mitochondrial genome from a specimen of the same species from China (NCBI Reference Sequence NC_021103.1) [23]. This was done using *BWA* with default settings, and coverage was estimated using BBMap [42] and SAMtools (version 1.4) [43]. Furthermore, the assembled transcripts were BLASTed against the reference mitochondrial genome to screen for potential chimeric components in the assembled transcripts. Variant calling was again performed using VarScan (version 2.4.2) [48] and all of the results were plotted and annotated using the Gviz package [49] on Bioconductor [50].

Finally, we aligned and estimated the sequence distance of four closely related Gomphocerinae mitogenomes, namely *G. rufus* (RefSeq: NC_014349), *G. licenti* (RefSeq: NC_013847), *G. tibetanus* (RefSeq: NC_015478), *G. sibiricus* from the Tianshan Mountians, Xinjiang, China (RefSeq: NC_021103) and our data from *G. sibiricus* from the European Alps. The multiple sequence alignment was estimated using *MAFFT* [51] with the default setting in ‘auto’ mode. The program *dnadist* from the *PHYLIP* package (version 3.696) [52] was used with default settings (F84 substitution model, transition/transversion ratio 2.0, using empirical base frequencies) to estimate pairwise sequence distances. The program *dnaml* from the PHYLIP package [52] was used to estimate the best fitting phylogenetic tree with default settings (transition/transversion ratio 2.0, constant rate variation among sites), with randomized input order of sequences, and *G. rufus* set as the outgroup and plotted (see Additional file 4: Figure S4).

## Additional files

Additional file 1: **Figure S1**. Log of average fold coverage versus log of contig length. (DOCX 58 kb)

Additional file 2: **Figure S2**. Top 20 taxa classes reported by BLASTX. (DOCX 50 kb)

Additional file 3: **Figure S3**. Classifications of GO terms of contigs. (DOCX 251 kb)

Additional file 4: **Figure S4**. A phylogeny based on mitochondrial sequences of four Gomphocerine grasshopper species. (JPG 35 kb)

Additional file 5: **Table S1**. A Summary table of the annotation of the contigs using the *dammit!* Pipeline. (DOCX 13 kb)

Additional file 6: **Table S2**. Sequence divergence matrix from four Gomphocerine grasshopper species. (DOCX 13 kb)

Additional file 7: **Table S3**. Read mapping statistics for non-rRNA reads. (DOCX 12 kb)

Additional file 8: Table of top Wolbachia BLASTX hits from the assembly. (CSV 104 kb)

## Abbreviations

BLAST: Basic local alignment tool
bp: base pair
BUSCO: Benchmarking Universal Single-Copy Orthologs
ERCC: External RNA Control Consortium
GO: Gene Ontology
KEGG: Kyoto Encyclopedia of Genes and Genomes
MAF: Minor Allele Frequency
ORF: Open Reading Frames
