# Supplementary figures and images for: Transcriptome assembly for a colour-polymorphic grasshopper (Gomphocerus sibiricus) with a very large genome size
Source: BMC Genomics. 2019 May 14;20:370. doi: 10.1186/s12864-019-5756-4 (PMC6518663; doi:10.1186/s12864-019-5756-4)

Figure S1: Log of average fold coverage versus log of contig length


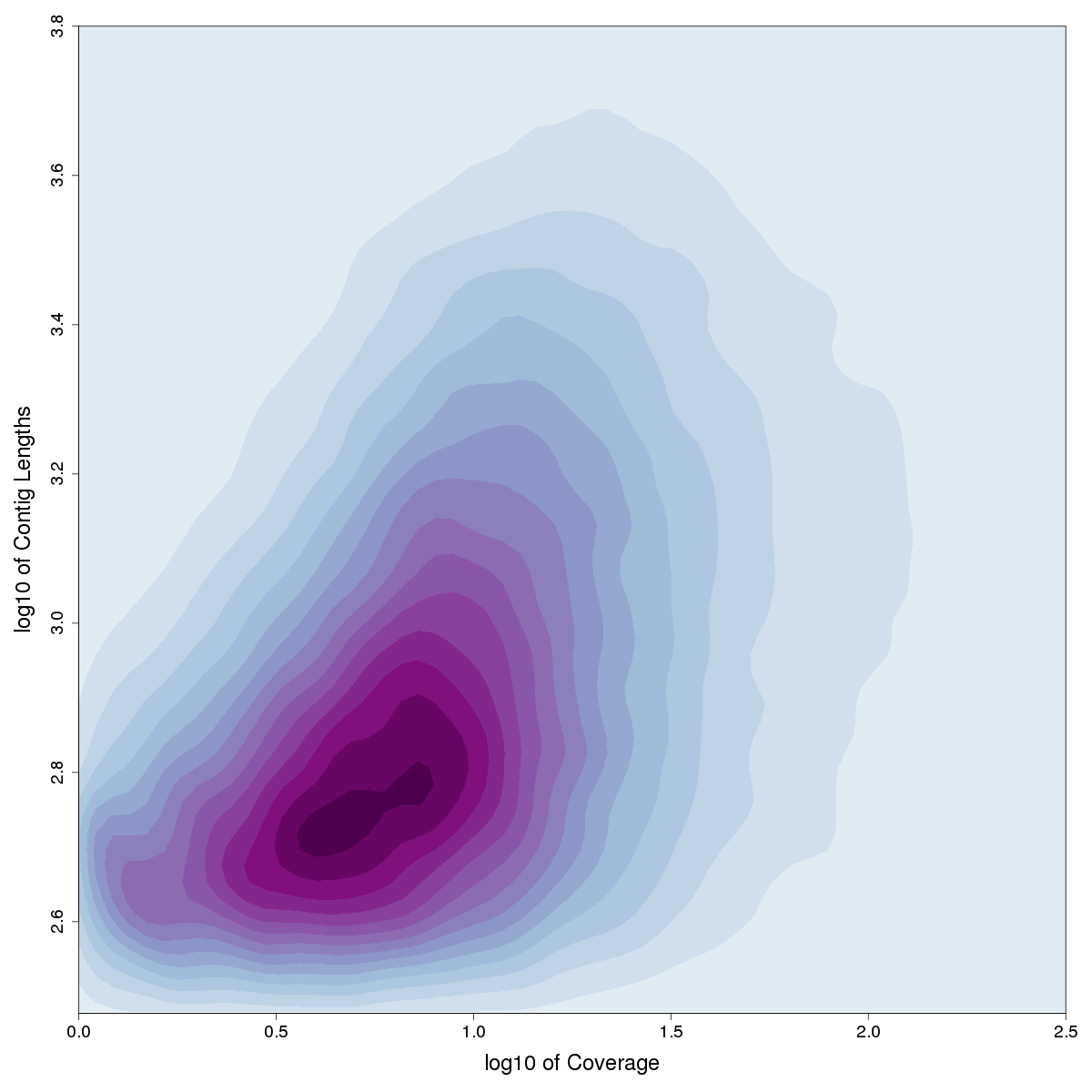

Supplement: Supplementary file 1 — Figure S1. Log of average fold coverage versus log of contig length. (DOCX 58 kb) [file 12864_2019_5756_MOESM1_ESM.docx]

Figure S2: Top 20 taxa classes reported by BLASTX.


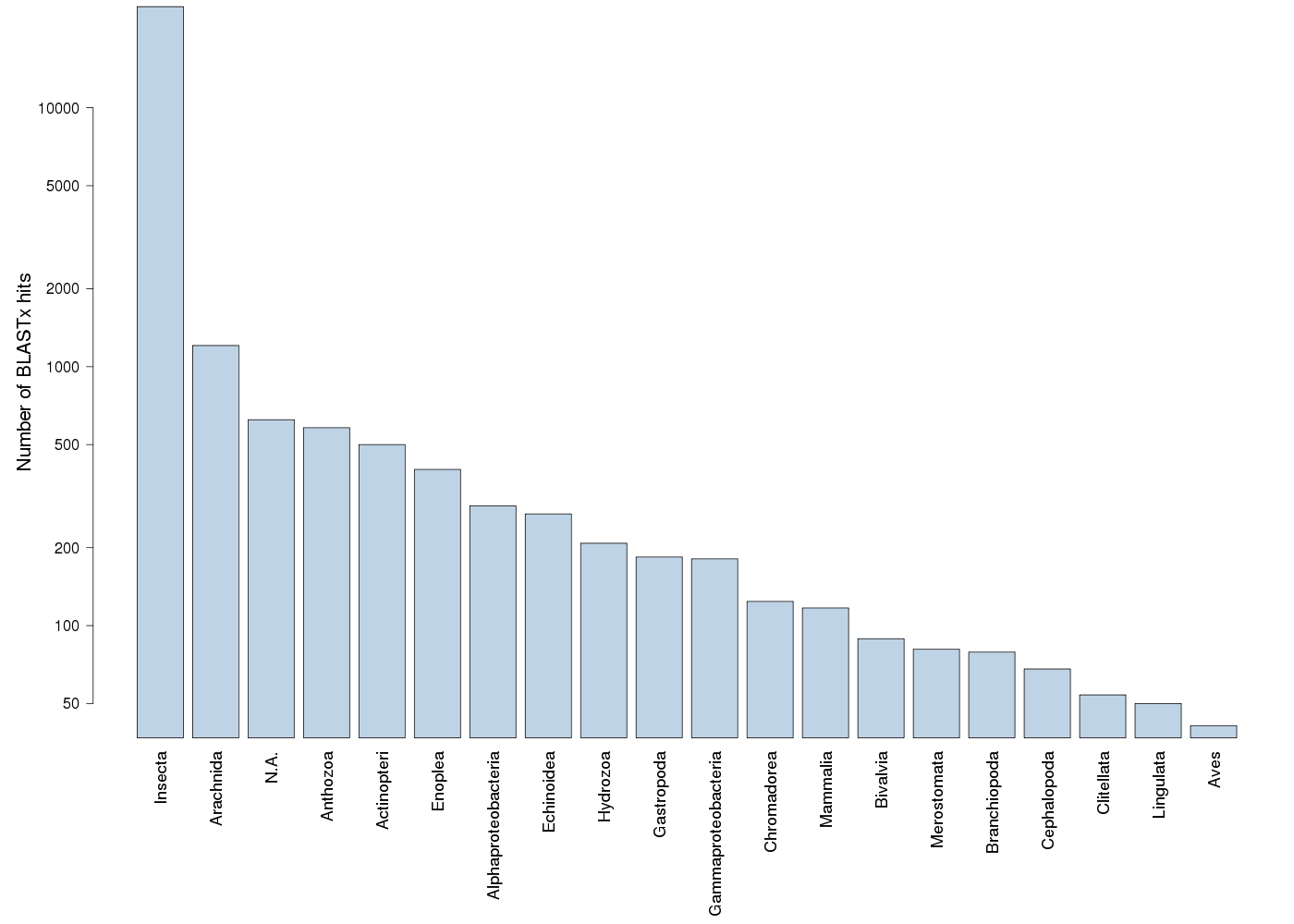

Supplement: Supplementary file 2 — Figure S2. Top 20 taxa classes reported by BLASTX. (DOCX 50 kb) [file 12864_2019_5756_MOESM2_ESM.docx]

Figure S3


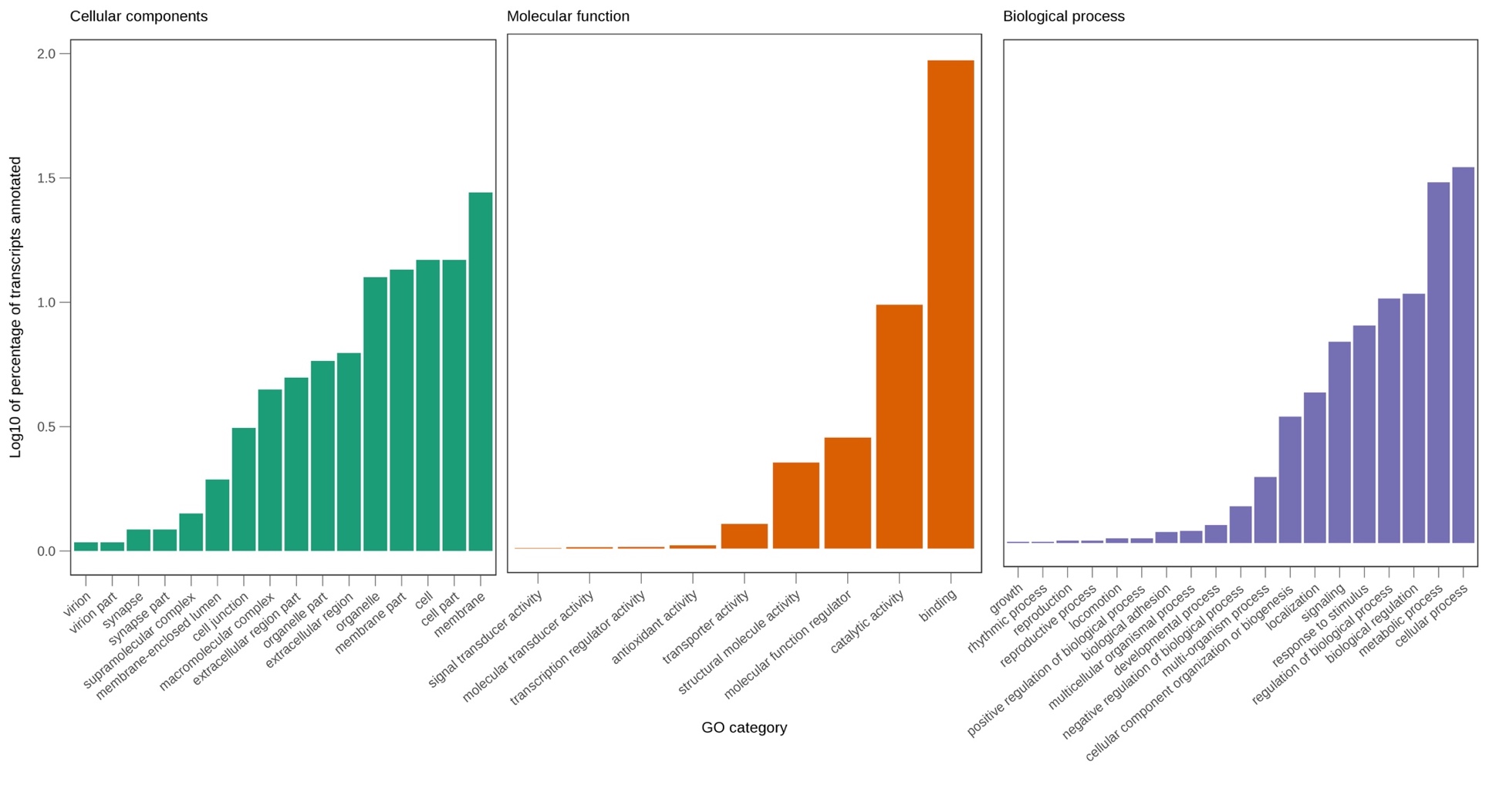

Supplement: Supplementary file 3 — Figure S3. Classifications of GO terms of contigs. (DOCX 251 kb) [file 12864_2019_5756_MOESM3_ESM.docx]

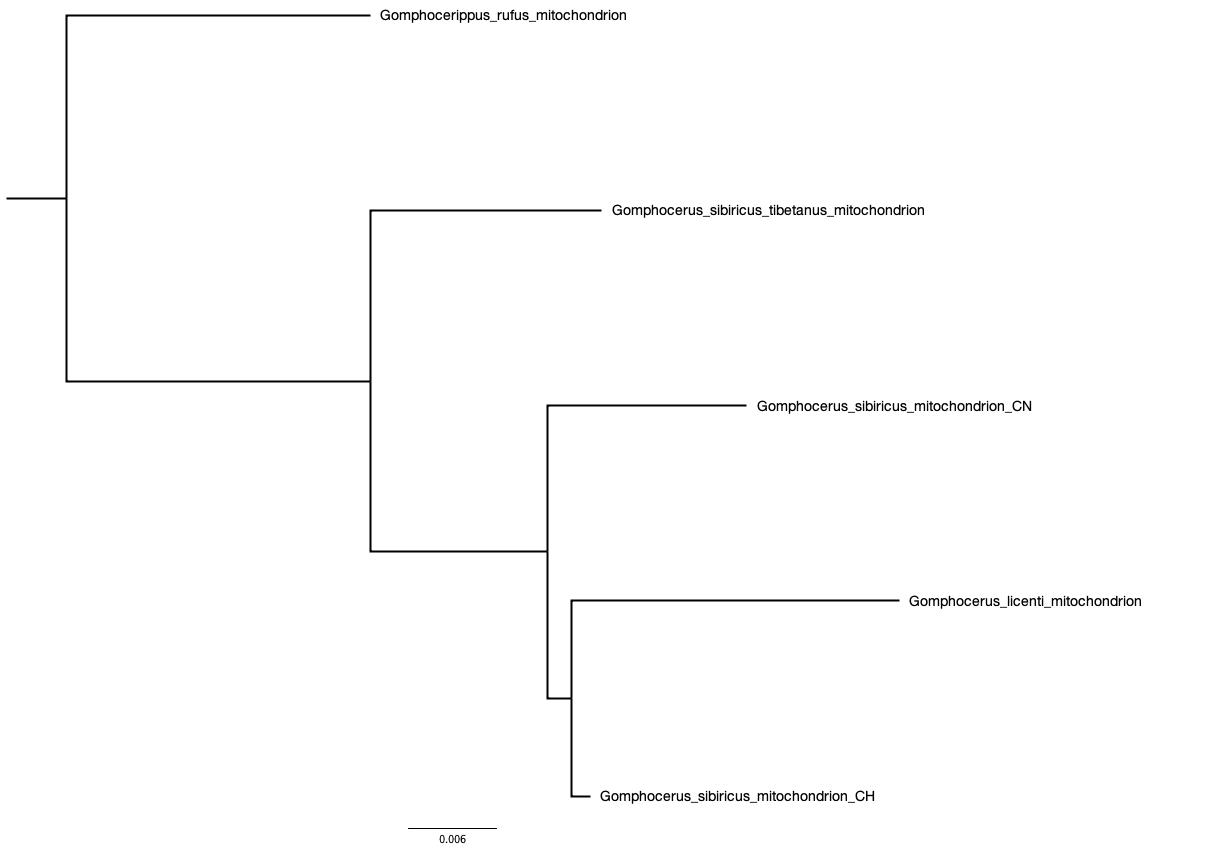

Supplement: Supplementary file 4 — Figure S4. A phylogeny based on mitochondrial sequences of four Gomphocerine grasshopper species. (JPG 35 kb) [file 12864_2019_5756_MOESM4_ESM.jpg]
