# Supplementary material for: Transcriptome assembly for a colour-polymorphic grasshopper (Gomphocerus sibiricus) with a very large genome size
Source: BMC Genomics. 2019 May 14;20:370. doi: 10.1186/s12864-019-5756-4 (PMC6518663; doi:10.1186/s12864-019-5756-4)
Supplement: Supplementary file 5 — Table S1. A Summary table of the annotation of the contigs using the dammit! Pipeline. (DOCX 13 kb) [file 12864_2019_5756_MOESM5_ESM.docx]

Table S1: Summary of the *dammit!* annotation. Contigs may be matched by multiple categories.

| Annotation | Contig count |
| --- | --- |
| Protein match | 65,665 |
| Translated nucleotide match | 71,206 |
| Exon | 39,056 |
| 3‘UTR | 35,203 |
| 5’UTR | 33,656 |
| mRNA | 39,056 |
| RNA sequence secondary structure | 1,264 |
| CDS | 39,056 |
