# Supplementary material for: Transcriptome assembly for a colour-polymorphic grasshopper (Gomphocerus sibiricus) with a very large genome size
Source: BMC Genomics. 2019 May 14;20:370. doi: 10.1186/s12864-019-5756-4 (PMC6518663; doi:10.1186/s12864-019-5756-4)
Supplement: Supplementary file 6 — Table S2. Sequence divergence matrix from four Gomphocerine grasshopper species. (DOCX 13 kb) [file 12864_2019_5756_MOESM6_ESM.docx]

Table S2: Matrix of sequence divergence across five mitochondrial sequences from four Gomphocerine species (*G. rufus*, *G. licenti*, *G. tibetanus*, *G. sibiricus* mitochondrial genome from this study (denoted by CH), and from Asia (denoted by CN)).

|  | *G. rufus* | *G. licenti* | *G. tibetanus* | *G. sibiricus* (CH) | *G. sibiricus* (CN) |
| --- | --- | --- | --- | --- | --- |
| *G. rufus* | - | 0.0718 | 0.0551 | 0.0533 | 0.0611 |
| *G. licenti* | 0.0718 | - | 0.0484 | 0.0233 | 0.0345 |
| *G. tibetanus* | 0.0551 | 0.0484 | - | 0.0291 | 0.0405 |
| *G. sibiricus* (CH) | 0.0533 | 0.0233 | 0.0291 | - | 0.0161 |
| *G. sibiricus* (CN) | 0.0611 | 0.0345 | 0.0405 | 0.0161 | - |
