# Supplementary material for: Transcriptome assembly for a colour-polymorphic grasshopper (Gomphocerus sibiricus) with a very large genome size
Source: BMC Genomics. 2019 May 14;20:370. doi: 10.1186/s12864-019-5756-4 (PMC6518663; doi:10.1186/s12864-019-5756-4)
Supplement: Supplementary file 7 — Table S3. Read mapping statistics for non-rRNA reads. (DOCX 12 kb) [file 12864_2019_5756_MOESM7_ESM.docx]

Table S3 :Read mapping statistics for non-rRNA cDNA reads and the complete cDNA library

|  | Non-rRNA cDNA library | Complete cDNA library |
| --- | --- | --- |
| Reads supplementary mapped | 318365 | 617983 |
| Reads mapped | 11469092 | 16024724 |
| Reads unmapped | 453289 | 671115 |
| Singletons | 145035 | 256251 |
| Total reads | 11922381 | 16695839 |
